# Thoracic Spinal Stenosis From Calcified Ligamentum Flavum

**DOI:** 10.31486/toj.23.0003

**Published:** 2023

**Authors:** Daniel R. Cavazos, Rebecca Schultz, Devan O. Higginbotham, Rahul Vaidya

**Affiliations:** ^1^ Department of Orthopaedic Surgery, Detroit Medical Center, Detroit, MI; ^2^ School of Medicine, Wayne State University, Detroit, MI

**Keywords:** *Calcification–physiologic*, *ligamentum flavum*, *spinal stenosis*, *spine*, *thoracic vertebrae*

## Abstract

**Background:** Calcification of the ligamentum flavum is a rare cause of spinal stenosis. The process can occur at any level in the spine, often presents with local pain or radicular symptoms, and is a distinct process from ossification of the spinal ligaments in pathogenesis and treatment approach. Few case reports have described multiple level involvement in the thoracic spine that results in sensorimotor deficits and myelopathy.

**Case Report:** A 37-year-old female presented with progressive sensorimotor deficits from T3 distally that resulted in complete sensory deficits and diminished lower extremity strength. Computed tomography and magnetic resonance imaging demonstrated calcification of the ligamentum flavum from T2-T12 with severe spinal stenosis at T3-T4. She underwent T2-T12 posterior laminectomy with ligamentum flavum resection. Postoperatively, she had complete motor strength return and was discharged home for outpatient therapy. Her residual sensory deficits continued to improve with time after decompression and excision of the calcified ligamentum flavum.

**Conclusion:** This case is unique in that the calcific process involved nearly the entire thoracic spine. The patient had dramatic improvement in her symptoms following resection of the involved levels. The case adds a severe manifestation of calcification of the ligamentum flavum with a surgical outcome to the literature.

## INTRODUCTION

Spinal stenosis is the narrowing of the spinal canal and compression of the nerve roots or spinal cord resulting in myelopathy or radiculopathy. Stenosis can involve the cervical, thoracic, or lumbar spine. Spinal stenosis can involve 3 regions: the central area, lateral recess, or neural foraminal region. The etiology of stenosis is either congenital or acquired. Acquired stenosis can be degenerative, traumatic, iatrogenic, or neoplastic in nature.^1,2^ Rare cases of stenosis derive from ossification of the posterior longitudinal ligaments in the cervical spine, a condition that is commonly reported in Japan.^3^ However, calcification of the ligamentum flavum is a separate entity from ossification of the posterior longitudinal ligaments^4^ and most commonly occurs in the cervical and lumbar spine.^5-9^ Few cases of calcification of the ligamentum flavum in the thoracic region have been reported.^10-12^ To our knowledge, no cases have reported calcification spanning nearly the entire thoracic spine. We report a case of spinal stenosis resulting from calcification of the ligamentum flavum, spanning T2 to T12, and resulting in extreme lower extremity weakness and sensory deficits.

## CASE REPORT

**Figure 1.**
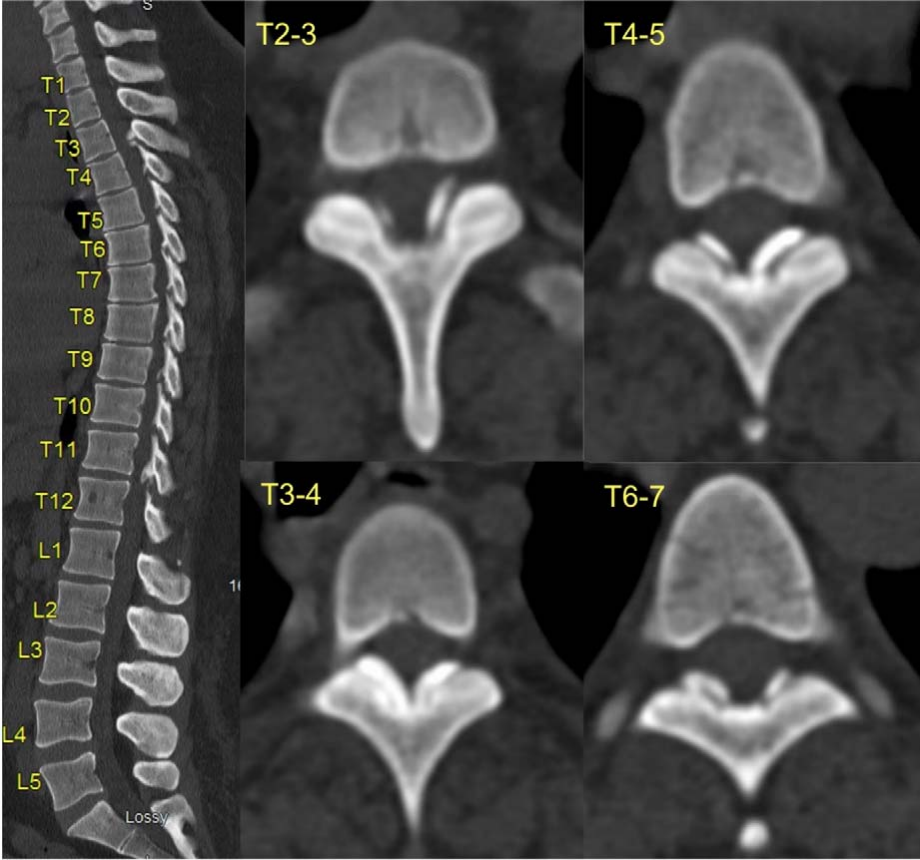
Sagittal (left) and axial (right) computed tomography cuts (T2-T7) of the thoracic spine showing segmental calcific lesions causing severe spinal stenosis.

A 37-year-old female presented to the emergency department (ED) directly from the neurology clinic for consultation for possible cord decompression. The patient reported that sensory changes from the nipple level down through the lower extremities with associated strength deficits had been developing for the prior 9 months. She had associated mid to lower back pain but no cervical pain. Three months prior to her presentation, the patient had a mechanical fall, and radiographs and computed tomography (CT) scans of her cervical, thoracic, and lumbar spine demonstrated no acute fractures and normal anatomic alignment. However, the CT scan of the thoracic spine showed calcification of the ligamentum flavum from T2-T12. Associated thoracic spinal stenosis was severe at T3-T4, moderate at T2-T3, and moderate at T4-T12 (Figure 1). The patient established care with neurology at this point, and her symptoms were conservatively managed with physical therapy and anti-inflammatory pain medication. However, during the months following her fall, she developed worsening sensory and motor deficits that caused her to be wheelchair-bound and unable to work as a cook.

**Figure 2.**
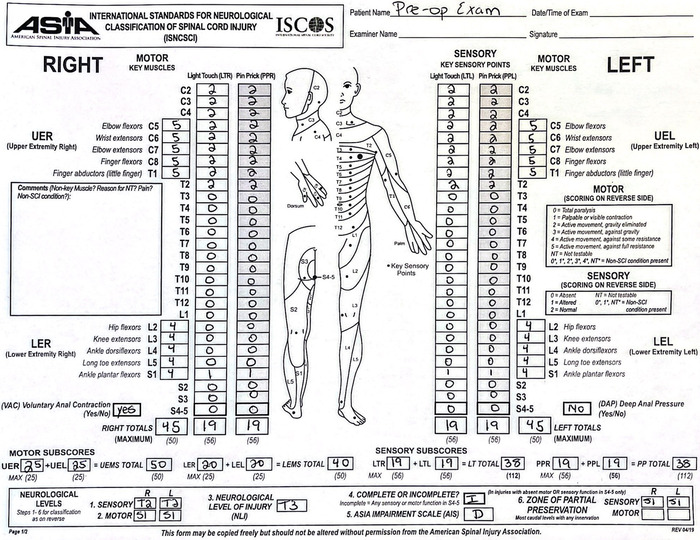
Preoperative American Spinal Injury Association (ASIA) assessment was consistent with an incomplete spinal injury of ASIA impairment scale level D at the level of T3. Cranial to T3, the patient had normal strength and sensation. Caudal to T3, she had no sensation aside from 1+ at S1 bilaterally. Her strength in the bilateral lower extremities was graded as 4 of 5 from L2-S1.

The patient's preoperative American Spinal Injury Association (ASIA) assessment (Figure 2) was consistent with an incomplete spinal injury of ASIA impairment scale level D at the level of T3. Cranial to T3, the patient had normal strength and sensation. Caudal to T3, she had no sensation aside from 1+ at S1 bilaterally. Her strength in the bilateral lower extremities was graded as 4 of 5 from L2-S1. She had voluntary anal contraction but no deep anal sensation. Her biceps, triceps, and Achilles reflexes were normal, but her patellar reflex was hyperreflexic bilaterally and she had an inverted brachioradialis reflex. She had negative clonus but positive Babinski and Hoffmann signs bilaterally. She had negative straight leg raises bilaterally. The patient could not be assessed for tandem gait because of weakness and was not ambulating on presentation. She could not tolerate a Romberg test.

**Figure 3.**
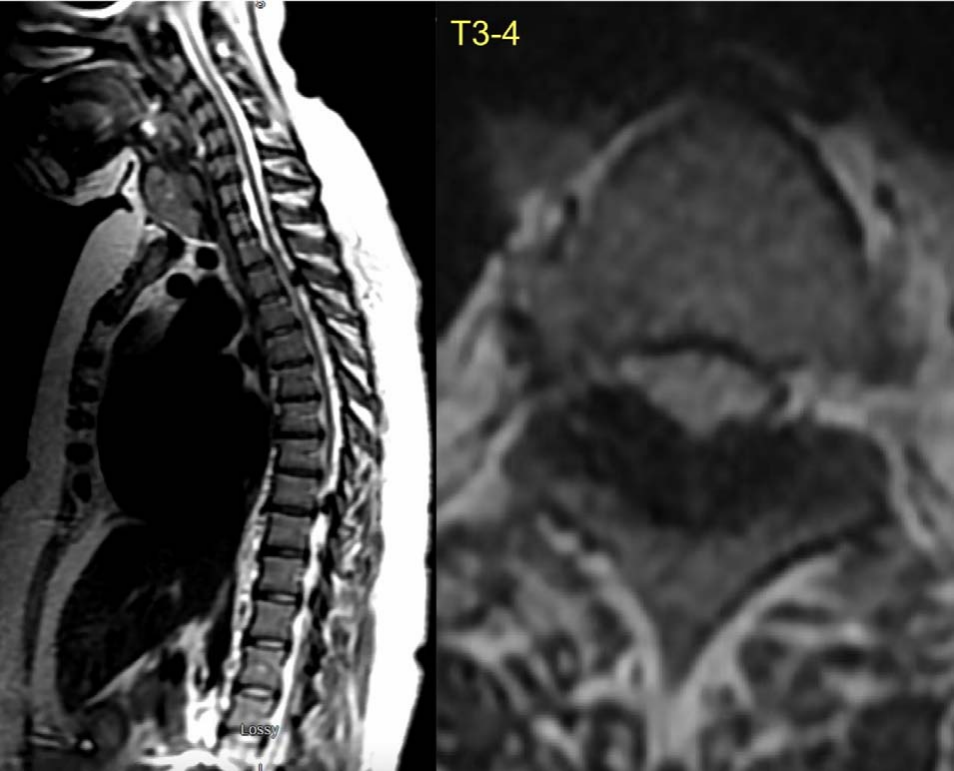
T2 magnetic resonance imaging cuts (left) showing multiple segmental calcifications of the ligamentum flavum throughout the thoracic spine, with severe spinal stenosis at the T3-T4 level (right).

Bilateral upper and lower extremity electromyography tests obtained in the neurology clinic prior to the patient's presentation to the ED were negative for any peripheral nerve lesions, inconsistent with her clinical picture. The neurologists were not concerned for any cranial or neuromuscular processes. Stat magnetic resonance imaging (MRI) studies without contrast were obtained of the cervical, thoracic, and lumbar spine. No pathology or lesions were seen at the cervical or lumbar levels. Thoracic-level MRI findings were consistent with the CT scan obtained earlier (Figure 3).

**Figure 4.**
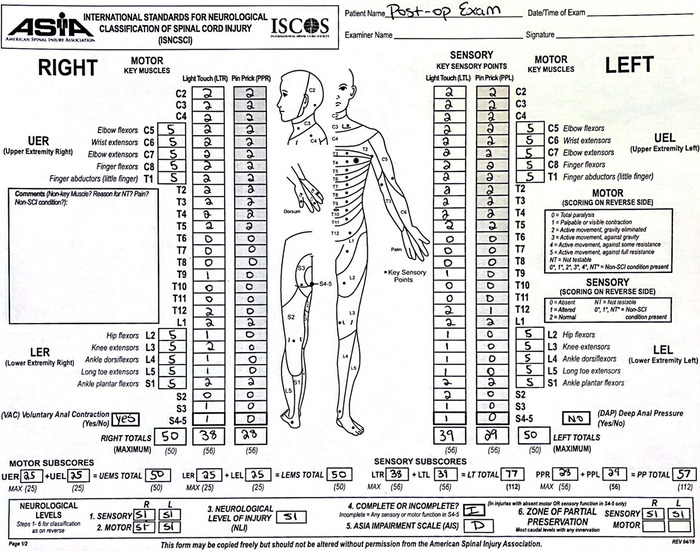
Postoperative American Spinal Injury Association (ASIA) assessment performed 1 week postoperatively was consistent with an incomplete spinal injury of ASIA impairment scale level D at the level of S1. The patient's bilateral lower extremity strength improved to 5 of 5. Sensory levels improved at multiple levels and have continued to improve with further follow-up.

Given that spinal stenosis was severe at T3-T4, moderate at T2-T3, and moderate at T4-T12, the decision was to perform a posterior laminectomy and resection of the calcified ligamentum flavum from T2-T12 without fusion. The patient's spinal cord was found to be significantly compressed from T2-T12 intraoperatively from calcified ligamentum flavum that was adherent to the dural layer. Intraoperative samples of the ligamentum flavum were sent to pathology, and the report was consistent with calcification and reactive bony processes. Postoperative ASIA assessment done on the day of the patient's discharge from the hospital 1 week postoperatively showed return of motor strength in her lower extremities to 5 of 5 and sensation return to both light touch and pinprick at multiple levels caudal to T3 (Figure 4). She was discharged home with outpatient therapy. She was seen at 2 weeks, 6 weeks, and 3 months postoperatively. At her 3-month follow-up, the patient was ambulatory with a walker and had improvement in her sensation below T10.

## DISCUSSION

Calcification of the ligamentum flavum is a rare and separate entity from ossification of the posterior longitudinal ligament. Ossification of the posterior longitudinal ligament involves ossification or bony formation of the posterior longitudinal ligament through endochondral bone formation. Ossification involves a process of laying down bone through osteoblasts.^9^ Our patient's pathology showed no such process but instead the development of calcium deposits. Calcification of the ligamentum flavum is a process of unknown pathogenesis.^4^ A proposed theory is that repetitive microtrauma to the ligamentum flavum initiates the calcific deposition. This microtrauma leads to neovascularization, permeability, and hypertrophy of the ligamentum flavum.^13,14^ The majority of cases report segmental involvement at just a few levels.^5-12^ Our case is unique in that our patient presented with multiple levels of involvement from T2-T12 and progressive myelopathy that eventually led to ambulatory deficits and severe sensorimotor deficits. To our knowledge, this case is the first report showing multiple levels of involvement of the thoracic spine with a myelopathic presentation.

Miyasaka et al stress the importance of assessment and monitoring through x-ray, CT, and MRI to differentiate ossification from calcification for clinical management.^15^ Ossification is often continuous with the lamina and often becomes adherent to the dura, making laminectomy and decompression difficult.^16^ Calcification of the ligamentum flavum is often missed or the diagnosis is delayed. Our case highlights the utility of surgical decompression for myelopathic symptoms when conservative management has failed.

## CONCLUSION

This case adds a severe manifestation of calcification of the ligamentum flavum with a surgical outcome to the literature. Our patient had moderate to severe calcification throughout the thoracic spine that led to sensorimotor deficits and myelopathic symptoms during a 9-month period prior to surgery. She had a near full recovery following decompression and resection of the calcified ligamentum flavum from T2-T12.

## Notes

*This article meets the Accreditation Council for Graduate Medical Education and the American Board of Medical Specialties Maintenance of Certification competencies for Patient Care and Medical Knowledge.*
